# Environmental impact assessment of nanofluids containing mixtures of surfactants and silica nanoparticles

**DOI:** 10.1007/s11356-022-21598-9

**Published:** 2022-07-01

**Authors:** Manuela Lechuga, Mercedes Fernández-Serrano, Francisco Ríos, Alejandro Fernández-Arteaga, Ramón Jiménez-Robles

**Affiliations:** Department of Chemical Engineering, Faculty of Sciences, University of Granada, Campus Fuentenueva s/n., 18071 Granada, Spain

**Keywords:** Silica nanoparticles, Aerobic biodegradability, Nonionic surfactants, Anionic surfactants, Emerging pollutants, Nanofluids

## Abstract

Due to widespread use of nanoparticles in surfactant-based formulations, their release into the environment and wastewater is unavoidable and toxic for biota and/or wastewater treatment processes. Because of concerns over the environmental impacts of nanofluids, studies of the fate and environmental impacts, hazards, and toxicities of nanoparticles are beginning. However, interactions between nanoparticles and surfactants and the biodegradability of these mixtures have been little studied until now. In this work, the environmental impacts of nanofluids containing mixtures of surfactants and silica nanoparticles were valuated. The systems studied were hydrophilic silica nanoparticles (sizes 7 and 12 nm), a nonionic surfactant (alkyl polyglucoside), an anionic surfactant (ether carboxylic acid), and mixtures of them. The ultimate aerobic biodegradation and the interfacial and adsorption properties of surfactants, nanoparticles, and mixtures during biodegradation were also evaluated. Ultimate biodegradation was studied below and above the CMCs of the individual surfactants. The interfacial and adsorption properties of surfactant solutions containing nanoparticles were influenced by the addition of silica particles. It was determined that silica nanoparticles reduced the capability of the nonionic surfactant alkyl polyglucoside to decrease the surface tension. Thus, silica NPs promoted a considerable increase in the surfactant CMC, whereas the effect was opposite in the case of the anionic surfactant ether carboxylic acid. Increasing concentrations of surfactant and nanoparticles in the test medium caused decreases in the maximum levels of mineralization reached for both types of surfactants. The presence of silica nanoparticles in the medium reduced the biodegradability of binary mixtures containing nonionic and anionic surfactants, and this effect was more pronounced for larger nanoparticles. These results could be useful in modelling the behaviour of nanofluids in aquatic environments and in selecting appropriate nanofluids containing nanoparticles and surfactants with low environmental impact.

**Graphical abstract:**

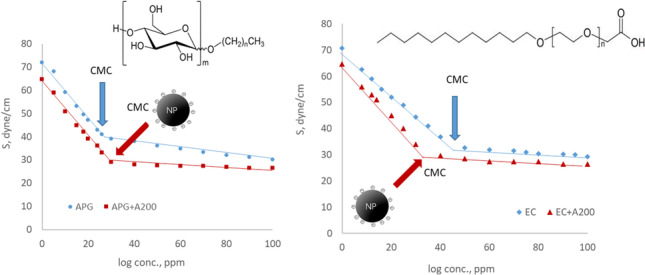

## Introduction

Despite the many applications and numerous advantages of surfactants in industrial and economic fields, from an environmental point of view, they are considered an important contaminant of aquatic environments, and high volumes of these substances are released daily into this medium. Once used, the surfactants reach treatment plants through urban and industrial wastewater and in certain cases are directly discharged into surface waters. During treatment of wastewater, a high percentage of the surfactants present in the aquatic environment are eliminated by aerobic biodegradation and adsorption onto particulate material, while the metabolites generated and the remaining nondegraded surfactants are dispersed in different environmental compartments. A growing problem is currently arising at wastewater treatment plants due to mixing of domestic wastewater and hospital and industrial effluent containing significant surfactant loads, and the mixtures may contain surfactants with different properties. The concentrations of surfactants present in domestic wastewater can vary between 1 and 10 mg/L, while they can reach levels of 300 mg/L in industrial wastewater (Siyal et al. 2020). Sewage treatment plants can lower the concentrations of surfactants by up to 1–3 mg/L, but surfactants are still present in active sludge, and this leads to significant environmental impacts (Bautista-Toledo et al. 2014).

The growing concern in recent years regarding the design of nonpolluting detergents has led to the development and use of more environmentally friendly surfactants, such as ether carboxylic acid derivatives and alkyl polyglucosides (APG) analysed in this study. The consumption of these surfactants is increasing year by year due to their remarkable environmental profiles. The fast-moving consumer goods industry demands products with low environmental impact, and consumers pay special attention to their components. Recent market reports (Fact.Mr. 2021) predict a growth of 0.6% in APG consumption this year, and through 2031, the market for APG is anticipated to expand at a high CAGR (compound annual growth rate) of close to 8%. This trend of replacing traditional surfactants with new biobased surfactants will continue to increase in the next few years. Therefore, a detailed study of new surfactants in combination with other surfactants and/or nanoparticles is mandatory for predicting their environmental impact.

The surfactant ether carboxylic acid is used in cleaning and cosmetic products that come in contact with the skin. These surfactants improve the foaming capacity of surfactant formulations and decrease levels of irritation (Jurado et al. 2011) when compared with other anionic surfactants. Alkyl polyglucosides have great advantages over other classes of surfactants. Their natural origin is the source of their good physical and environmental properties. Moreover, alkyl polyglucosides present high compatibility and foam production, excellent cleaning efficiency, wettability, and ocular and dermatological safety and have been proven to be readily biodegradable under aerobic conditions (Jurado et al. 2002; Zgoła-Grześkowiak et al. 2008). All of this makes them potential components for a variety of domestic and industrial applications (Pantelic and Cuckovic 2014; Tasic-Kostov et al. 2014).

The special properties of small particles (1 nm to 1 μm) and the advantages they offer in processes related to catalysis, new materials, or biomedicine have led to increased use in consumer products such as detergents (Ma et al. 2008). Scientific interest in recent years has focused on silica nanoparticles (Slowing et al. 2010; Mamaeva et al. 2013), and several detergent formulations and related formulations containing silica particles have been patented (Orlich et al. 2007). Nanoparticles are present in many formulations and applications due to their physicochemical properties, low toxicity, stability, and functionalization capacity with a range of polymers and molecules (Ríos et al. 2018a). Silica nanoparticles are frequently mixed with surfactants for oil recovery, nanofluid production, immobilization of enzymes, and removal of dyes, detergents, or foam stabilizers (Maestro et al. 2014; Zhu et al. 2015; Patra et al. 2016; Plomaritis et al. 2019).

As with surfactants, particles of colloidal size can accumulate spontaneously at liquid–gas or liquid–liquid interfaces where they are acting as stabilizers of emulsions and foams (Eskandar et al. 2011). Simple algorithms have recently been used to estimate potential concentrations of NPs from consumer products. However, the concentrations estimated by applying these models are significantly lower than the results of many published studies (Tiede et al. 2009). When nanoparticles are used together with surfactants, synergistic effects can be observed in the production of emulsions and stable foams, so it is of great interest to study these interactions from an environmental point of view.

Due to the widespread use of nanoparticles in formulations in recent years, their release into the environment and wastewater is unavoidable (Huang et al. 2017) and brings toxicity to biota and/or wastewater treatment processes. Because of increasing concern about the environmental impacts of the latest materials, studies of the toxicity, hazards, fate, and environmental impact of nanoparticles are beginning (Liu et al. 2014; Skorochod et al. 2016; Ríos et al. 2018b).

The interactions between nanoparticles and surfactants as well as the biodegradability of surfactant mixtures have not been sufficiently studied until now. A recent paper by Bimová et al. (Bimová et al. 2021) summarized the possible toxic effects of nanomaterials on the environment and living organisms due to their use in different technologies, environmental sectors, and medicine. However, this work did not include any reference to the mixtures of nanoparticle surfactants. From our humble point of view, this is consistent with the lack of knowledge in this particular field. Predictability of the joint effects of solutions containing surfactants and nanoparticles is of great interest for adequate assessments of environmental risk due to the growing usage of nanoproducts, nanomaterials, and nanofluids.

Biodegradability tests can produce variable results attributable to changes in inoculum, inoculum origin, and ratio, which result in false negatives (Lundgren et al. 2013). In this sense, “positive” results can be considered sufficient evidence of biodegradability and can generally be substituted for negative results. The OECD 301 series of readily biodegradable tests is considered the standard for screening purposes (OECD 1992). Ready biodegradability tests are conservative in nature and stringent enough to assume rapid and complete biodegradation of compounds in aquatic environments (OECD 1992).

This work is focused on biodegradation of anionic and nonionic surfactants, and their relative risk profiles are compared to those for mixtures of surfactants and surfactant-nanoparticles due to the high production volumes and the massive and dispersed use of surfactant-based formulations. The aerobic biodegradability of nanofluids, solutions containing silica nanoparticles in combination with an anionic surfactant (ether carboxylic acid), a nonionic surfactant (alkyl polyglucoside) whose individual environmental impacts have been previously assessed (Jurado et al. 2013; Lechuga et al. 2016; Ríos et al. 2017), and mixtures of them have been studied. In addition, with the goal of gaining insight into environmental behaviour and other aspects related to interfacial phenomena and cleaning efficiency, the effects of nanoparticles on the surfaces, interfacial tensions, and critical micellar concentrations (CMCs) of surfactants and mixtures were measured.

## Materials and methods

### Silica nanoparticles

**Fig. 1:**
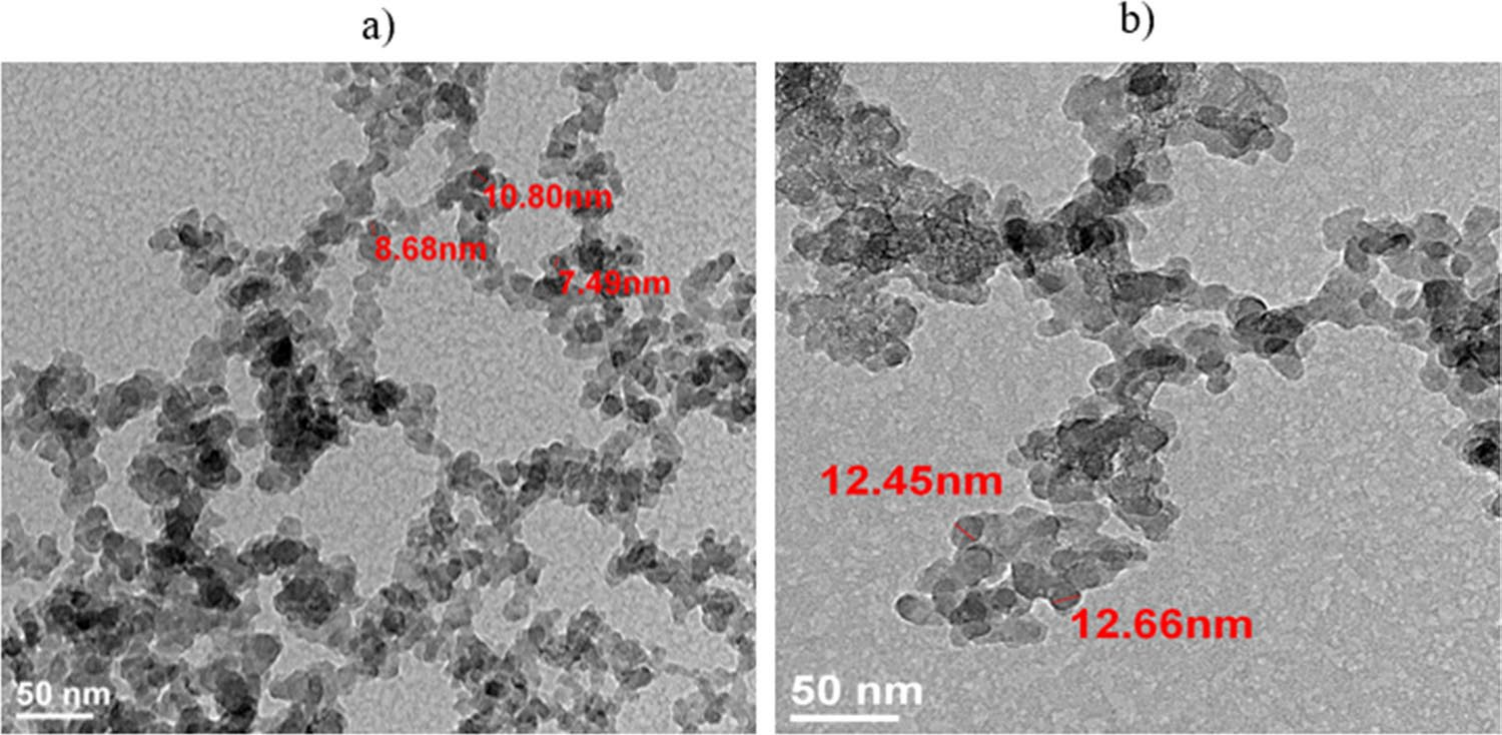
TEM images of nanoparticles: **a**) A380 and **b**) A200

Two types of hydrophilic silica nanoparticles (Aerosil 380 and Aerosil 200, Evonik Industries AG (Essen, Germany)) were used. Table 1 shows the physicochemical properties of the nanoparticles used in this study, including mean diameter (D_m_), specific surface area (S), tapped density (d), and pH. Nanoparticles were observed by TEM using an ultrahigh-resolution scanning transmission electron microscope (S/TEM) and a high-angle annular dark-field imaging (HAADF) system (FEI TITAN G2 60-300). The images showed amorphous structures for both nanoparticles, and these tended to be spherical in shape (Fig. 1), but both Aerosil 380 and Aerosil 200 showed sphericity values of 0.851 and 0.943, respectively. TEM analyses were performed to corroborate this statement.

**Table 1:** Physicochemical properties of nanoparticles

Nanoparticle	Abbr.	D_m_, nm	S , m^2^/g	d , g/L	pH value
Aerosil® 380	A380	7	380 ± 30	50	6.24−6.72
Aerosil® 200	A200	12	200 ± 25	50	6.14–6.54

### Surfactants

The nonionic surfactant alkyl polyglucoside (APG) was supplied by Sigma-Aldrich (St. Louis, USA), and the anionic surfactant ether carboxylic acid (EC) was provided by KAO Corporation (Tokyo, Japan). Table 2 summarizes their main characteristics. Surfactant solutions were studied at two concentrations, 25 mg/L and 50 mg/L. A binary mixture of these surfactants with a proportion of 1:1 (w/w) was also studied at a total concentration of surfactant of 50 mg/L.

**Table 2:** Details of surfactants used in the tests

Surfactant	EC-n_12-14_E_3_	APG-n_8-14_m_1.3_
Chemical name	Laureth-4 carboxylic acid	Coco glucoside
Trade name	AKYPO® RLM-25	Glucopon 650
Structure		
Active matter,^a^ %	93.1	48.6

^a^Determined using infrared radiation (Ríos et al. 2016)

*n*: Length of the alkyl chain, n-C_i_H_2i+1_-

*m*: Mean number of glucose units per surfactant molecule

### Sample preparation

A magnetic stirrer was used to wet the silica particles with aqueous media, and then dispersion and deagglomeration were performed by ultrasonication for 30 min (Sonorex RK 106 S, Bandelin, Berlin, Germany) in 1 L of ultrapure water. Subsequently, the surfactant was aggregated to obtain a suitable concentration. Ultrasonic cavitation helped to disperse particles since it generates high shear that breaks particle agglomerates. The interfacial tension, superficial tension, and biodegradability of surfactant solutions with silica nanoparticles were assessed as described in the following sections.

### Surface and interfacial tension

Surface and interfacial tensions were determined for nanoparticles and surfactants. Additionally, during the biodegradability tests, surface and interfacial tension were determined over time. Surface tension was measured at 25 °C using the Wilhelmy plate method with a Krüss KSV tensiometer equipped with a 2-cm platinum plate (Krüss GmbH, Hamburg, Germany). The platinum plate was cleaned by heating it to a reddish orange colour with a burner prior to use. Standard deviations were calculated by carrying out successive measurements, resulting in values less than 0.1 mN/m. The interfacial tensions (IFT) between dodecane and aqueous solutions were determined at 25 °C by a pendant drop tensiometer (KSV CAM 200, KSV Instruments Ltd, Finland). Measurements were performed in triplicate.

The critical micellar concentration CMC was calculated by plotting the surface tension vs. surfactant concentration (0 to 5·10^3^ mg/L). The break point in the plot indicates the formation of micelles. CMC results for anionic and nonionic surfactants are shown in Table 3.

**Table 3:** Surface and interfacial tensions of solutions

	Nanoparticle (mg/L)	Surfactant (mg/L)	Surface tension (mN/m)	Interfacial tension, (mN/m)	CMC (mg/L)
A380					
	0	--	72.28	43.41	--
	250	--	70.35	43.51	--
	1000	--	71.38	44.04	--
A200					
	0	--	72.28	43.41	--
	250	--	72.01	42.97	--
	1000	--	71.32	43.48	--
APG-R_8-14_DP_1.3_					29.08
	--	25	65.20	31.06	
	--	50	44.89	24.88	
EC-R_12-14_E_3_					33.24
	--	25	34.31	35.99	
	--	50	35.11	30.24	
EC-R_12-14_E_3_+APG-R_8-14_DP_1.3_					44.76
	--	25	52.46	32.13	
	--	50	42.05	27.47	
EC-R_12-14_E_3_+A200					
	250	50	33.80	31.71	28.40
	1000	50	28.30	23.85	27.92
EC-R_12-14_E_3_+A380					
	250	50	34.39	30.05	26.71
	1000	50	29.96	23.37	25.48
APG-R_8-14_DP_1.3_+A200					
	250	50	49.93	26.87	32.74
	1000	50	49.95	27.08	31.63
APG-R_8-14_DP_1.3_+A380					
	250	50	48.29	27.63	31.82
	1000	50	49.86	28.03	31.24
EC-R_12-14_E_3_+APG-R_8-14_DP_1.3_+A200					
	250	50	30.40	35.50	38.42
	1000	50	35.10	36.20	42.15
EC-R_12-14_E_3_+APG-R_8-14_DP_1.3_+A380					
	250	50	31.30	48.50	45.27
	1000	50	29.00	47.60	46.32

### Biodegradation and adsorption tests

Ultimate ready biodegradability tests followed OECD 301E test guidelines (OECD 1992). Ready biodegradability was determined for solutions containing individual and mixtures of surfactants. Reference assays were used as a positive control with a readily biodegradable surfactant (linear alkylbenzene sulfonate) to check the activity of the microbial population present in the test medium. The biodegradation tests are based on the removal of organic compounds measured as dissolved organic carbon (DOC) (OECD 2001). This test is quite rigorous due to the relatively high concentration of surfactant, the only carbon source, and the low proportions of inoculum and test compound. All experiments were performed at 25 °C. In biodegradation tests, surfactant solutions with nanoparticles were unique carbon sources for microorganisms. Surfactant and the nanoparticle solutions were prepared in a mineral medium, which was inoculated and incubated under aerobic conditions in darkness for 28 days. This mineral medium was prepared adding in 1 L of Milli-Q® water: 85.0 mg KH_2_PO_4_, 217.5 mg K_2_HPO_4_, 334.0 mg Na_2_HPO_4_.2H_2_O, 5.0 mg NH_4_Cl, 27.5 mg CaCl_2_, 22.5 mg MgS0_4_.7H_2_0, and 0.25 mg FeCl_3_.6H_2_0. The test medium was previously aerated for 30 min with carbon dioxide-free air and prepared according to guidelines for glass-distilled water and mineral salts. Duplicate tests were run for each test solution, along with the positive control and blank samples. The solution for which biodegradability was to be determined was inoculated with 0.5 mL of fresh activated sludge inoculum obtained from a municipal wastewater treatment plant that operates with active sludge (Granada, Spain, 37°09′54.1″N–3°37′31.8″W); this plant was selected because it deals predominantly with nonindustrial, municipal wastewater. This water sample was a mixed aerobic culture of faecal microorganisms, including, for the most part, total coliforms, faecal coliforms, and enterococcus. The microbial activity of the supernatant was determined to be 10^5^ to 10^6^ CFU/mL. Supernatant microbial sludge was added to the test medium.

Biodegradation was determined from the residual surfactant concentration over time by measuring dissolved organic carbon (DOC) in samples filtered through a 0.45-μm Millipore membrane. In the reference tests, the initial concentration of surfactant was 5 mg/L in all cases, and the average biodegradability reached at the end of the test was 98.34%; this fulfilled the 90% criterion set by the OECD for 5 days for soft standards and thus indicated the validity of the assay.

Test surfactant concentrations ranged from 25 to 50 mg/L in order to ensure at least 40 mg ThOD/L (Theoretical oxygen demand). The test temperature was maintained at 25 °C ± 1 °C (with minor deviations of less than 1 °C). All test vessels were stirred constantly with magnetic stir bars at 125 sweeps/min. All glassware was cleaned using a solution of ammonium persulfate in H_2_SO_4_ (98%).

Adsorption experiments were carried out under the same conditions as biodegradation tests but in the absence of microorganisms.

## Results and discussion

### Surface and interfacial tension

Surface and interfacial tensions of nanoparticle dispersions in Milli-Q® water were determined in the concentration range 0−1.000 mg/L at 25 °C. For both nanoparticle dispersions, the surface and interfacial tensions did not change with concentration, and the values were approximately 44.6 ± 0.4 mN/m for interfacial tension and 71.3 ± 0.6 mN/m for surface tension (Table 3), which are close to those between dodecane and pure water. Therefore, the silica particles were not surface active, and they did not show a preference for the water-air/dodecane interface due to their hydrophilic character. These results are consistent with the surface tension data obtained by Ma et al. (Ma et al. 2008) and Vatanparast et al. (Vatanparast et al. 2018) for Levasil® silica solutions.

Anionic and nonionic surfactants decrease the surface tensions of air–water interfaces and the interfacial tensions of liquid–liquid interfaces. As shown by the results in Table 3, the inclusion of negatively charged hydrophilic silica nanoparticles (diameters of 7–12 nm) in surfactant solutions modified their interfacial properties. Due to the assumed lack of surface-active character for silica nanoparticles, the differences in interfacial properties relative to those of the single surfactant system were attributed to nanoparticle-surfactant interactions (Vatanparast et al. 2018). In the case of anionic surfactants, silica nanoparticles increased the surface activity and therefore the efficiency of the EC surfactants due to repulsive coulombic interactions between the surfactants and nanoparticles, which promoted surfactant adsorption at air–water interfaces. Similar results were found by Ma et al. (Ma et al. 2008) for systems with SDS involving nanoparticles with diameters of 13 nm. For solutions involving nanoparticles and APG nonionic surfactants, which effectively decrease the efficiency and increase interfacial tensions, the nanoparticle effects were similar to those for air–water interfaces.

The surface and interfacial tensions for solutions of surfactant containing nanoparticles were measured, and CMCs were determined at 25 °C. Solutions containing nanoparticles and nonionic surfactant (APG) showed a CMC larger than that of pure surfactant solution, whereas solutions containing nanoparticles and anionic surfactant (EC) showed considerably reduced CMCs. Similar results were obtained by Rios et al. (Ríos et al. 2018a) for anionic-nonionic surfactant systems and silica nanoparticles.

**Fig. 2:**
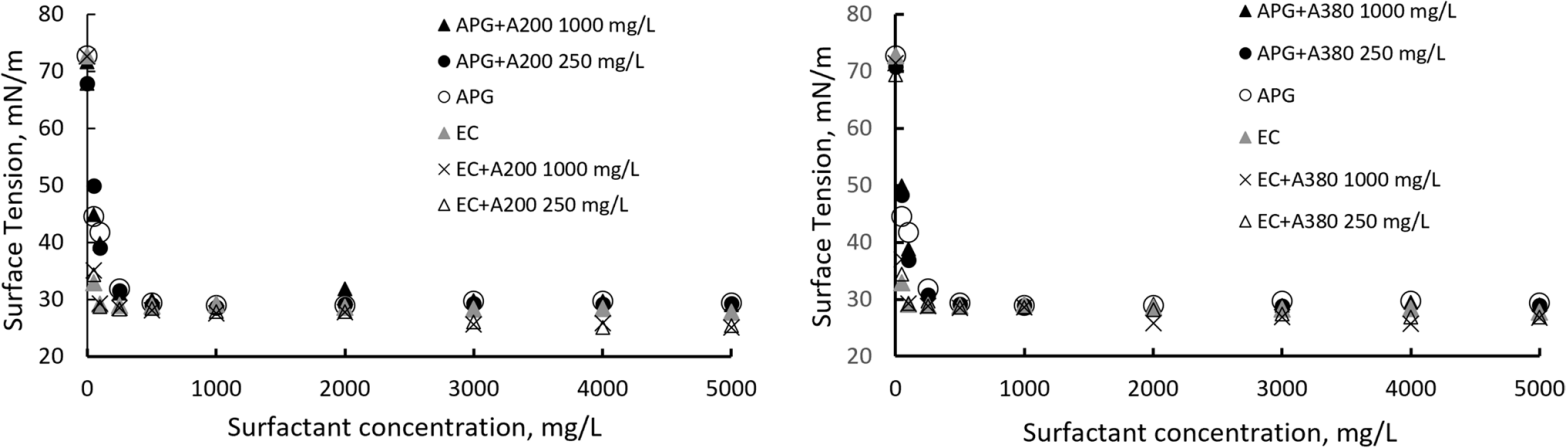
Surface tensions of nanofluids versus surfactant concentration

The modifications of surface and interfacial tensions were the same when using the same surfactant with different nanoparticles, and CMCs were similar or on the same order of magnitude (Fig. 2). The decrease in CMC with anionic surfactant was due to repulsive electrostatic forces operating between particles with anionic surfactant that favour diffusion of surfactant molecules towards the interface (Zargartalebi et al. 2015). Silica particles make the Gibbs free energy for adsorption and micellization more negative (Ma et al. 2008), which promotes adsorption and aggregation in micelles. The decrease in CMC was greater in the case of anionic surfactant solutions containing smaller nanoparticles. In the case of a nonionic surfactant, the effect was opposite because adsorption and electrostatic forces were much weaker; in this case, micellization and effects on Gibbs free energy were negligible.

### Biodegradability of surfactants and silica nanoparticles

**Fig. 3:**
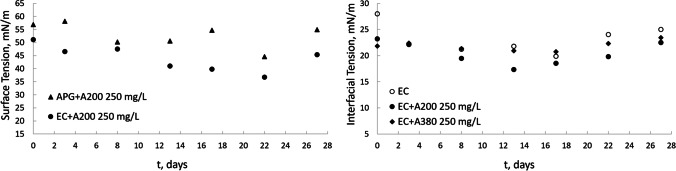
Surface and interfacial tensions of nanofluids during adsorption tests. The initial surfactant concentration was 25 mg/L

The aerobic biodegradation of ether carboxylic acid and alkyl polyglucoside solutions in combination with 250 mg/L hydrophilic fumed silica nanoparticles was studied. The initial surfactant concentrations in the biodegradability tests were below or above CMC, 25 and 50 mg/L. Surfactant adsorption onto materials considerably influences the environmental impact of surfactants, and some authors have studied this phenomenon (Belanger et al. 2006; van Compernolle et al. 2006). Adsorption tests were carried out in experiments with anionic and nonionic surfactants and mixtures of these surfactants with A200 and A380 nanoparticles. During the tests, the presence of surfactant was determined by DOC measurements. Additionally, the surface and interfacial tensions were analysed during the entire adsorption experiment (Fig. 3). Abiotic tests were carried out with dilute HgCl_2_ to confirm adsorption, and it was found that the residual concentrations of surfactant remained at approximately 99% during the biodegradation period. The surface and interfacial tensions were approximately constant, which confirmed that there was no adsorption of the surfactants on the nanoparticles. This fact was observed independently of either the ionic character of the surfactant or the nanoparticle size. Thus, in the adsorption tests presented in this work, the results indicated that the contribution of the abiotic process degradation of the surfactant can be neglected even in the presence of nanoparticles. For solutions with nanoparticles and surfactants, the surface and interfacial tensions were lower for larger nanoparticles. If several adsorption experiments are compared according to surfactant character, it is observed that the interfacial and surface tensions were lower for the anionic surfactant even in the presence of nanoparticles, indicating less adsorption of anionic surfactants on nanoparticles compared with nonionic surfactants. This can be explained by electrostatic repulsion between the anionic surfactant and hydrophilic silica nanoparticles, which are negatively charged.

**Fig. 4:**
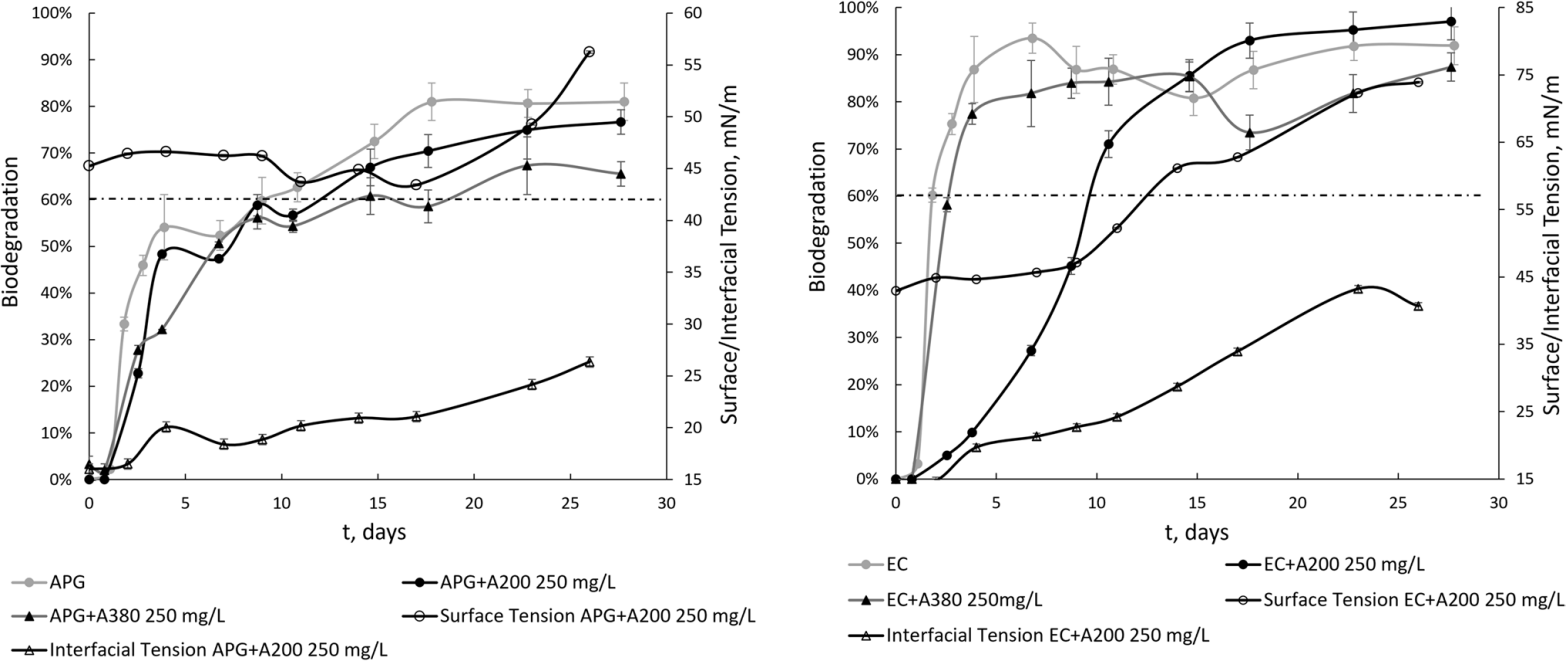
Time course for biodegradation of APG and EC. Initial surfactant concentrations were 25 mg/L

Figure 4 shows the time course of biodegradability over the degradation period for solutions of APG and EC with initial surfactant concentrations of 25 mg/L. The tests were carried out on surfactant solutions without nanoparticles (25–50 mg/L surfactant solutions) and solutions of surfactant containing nanoparticles at concentrations of 250 mg/L. The results showed that the effects produced by nanoparticles were highly dependent on the initial surfactant concentration in the test medium. Generally, the results showed that the presence of nanoparticles reduced primary and final biodegradation. This reduction in biodegradability of the anionic surfactant due to the presence of nanoparticles was 7.06% when the concentration of nanoparticles was increases from 0 to 250 mg/L and 10.67% for nonionic surfactants. Regardless of the presence of nanoparticles in the solutions, the anionic surfactant was more biodegradable than the nonionic surfactant. Table 4 shows that EC and APG were readily biodegraded. A surfactant can be considered biodegradable if one of the tests indexed in Annex III of Regulation (EC) No. 648/2004 (Regulation (EC) 2004, 2008) exhibits a minimum ultimate biodegradation level of 60% after 28 days. The surfactants EC and APG fulfilled this requirement and yielded 91.8% and 80.64% DOC removal, respectively, for initial concentrations of 25 mg/L and 80.15% and 60.49% for initial concentrations of 50 mg/L.

**Table 4:** Characteristic parameters for biodegradation profiles

	t_L,_ (h)	t_1/2,_ (h)	V_M_ (%/h)^(a)^	B (%)	Min (%)^(b)^
APG-R_8-14_, 25 mg/L	15.12	117.36	0.17	25.60	80.64
EC-R_12-14_, 25 mg/L	16.32	52.32	0.83	46.50	91.8
APG-R_8-14_, 25 mg/L. A200, 250 mg/L	15.96	122.52	0.29	17.54	76.66
APG-R_8-14_, 25 mg/L. A380, 250 mg/L	21.60	177.24	0.23	25.04	65.53
EC-R_12-14_, 25 mg/L. A200, 250 mg/L	78.24	206.64	0.28	2.55	87.02
EC-R_12-14_, 25 mg/L. A380, 250 mg/L	21.18	46.64	0.57	57.12	77.38

^(a)^Calculated for 50% biodegradability

^(b)^Data for the ultimate biodegradation profiles

Surface and interfacial tensions were also determined for solutions containing nanoparticles during the biodegradation process. As the biodegradation process proceeded, increases in surface and interfacial tensions confirmed the disappearance of surfactant from the medium, which was consistent with the degradation curves obtained (Fig. 4). In the case of the nonionic surfactant (APG), increases in the superficial and interfacial tensions during biodegradation were smoother than those for the anionic surfactant, which may have arisen because the degradation metabolites of APG have a certain interfacial activity. APG follows a central scission biodegradation pathway in which ω-oxidation and central scission lead to dicarboxylic acids (Jurado et al. 2011), and the interfacial activity is associated with this process (Lee and Hildemann 2013). Regardless of the presence of nanoparticles in the biodegradation tests, it was observed that the anionic surfactant had higher surface and interfacial activities than the nonionic surfactant. This is directly related to low adsorption of the anionic surfactant during nanoparticle adsorption tests and the higher biodegradability of EC compared to APG. The higher hydration capacity of the polar head in the anionic surfactant makes adsorption more difficult than it is for nonionic surfactants. For APG, the adsorption of surfactant onto nanoparticles drove the nanoparticles towards the interfaces due to the increased hydrophobicity (Ravera et al. 2006). On the other hand, APG formed suspensions, and only a small part of the surfactant may be susceptible to biodegradation and available to bacteria (Zgoła-Grześkowiak et al. 2008), consistent with the lower biodegradation relative to that of EC.

When comparing the influence of nanoparticle size on the biodegradability, it was observed that larger particles caused greater biodegradability independent of the character of the surfactant (Fig. 4). Both surfactants decreased the diameters of nanoparticle aggregates and increased their effective concentrations. To corroborate this, parameters characteristic of biodegradation profiles (Jurado et al. 2007) were calculated, including latency time (t_L_), half-life time (t_1/2_), mean biodegradation rate (V_M_), percentage of primary biodegradation reached at 50 h of assay (B), and mineralization (Min). Table 4 summarizes the values of these characteristic parameters obtained for the biodegradation profiles. Equations 1 and 2 show the dependence of biodegradation (B) and mean biodegradation rate on nanoparticle concentration for APG and A200 nanoparticles.

1 $$Vm=0.159.{e}^{0.0015}\left[A200\right]$$

2 $$B=22.22.{e}^{-0.001}\left[A200\right]$$

The nanoparticles affected the acclimation time of the microorganisms, t_L_; this value varied between 15.96 h for the APG-A200 assay and 78.24 h for the EC-A200 assay. The latency time and half-life time were notably augmented for nanofluids containing A200 nanoparticles. The presence of nanoparticles in the biodegradability test did not alter the form of the resulting curve except for anionic surfactant EC and A200 nanoparticles, the shape of the curve was exponential, the biodegradation process became slower (B = 2.55%), and a long lag phase was observed, although the final mineralization level reached was the highest among the tests carried out with nanoparticles (Min = 87.02%). Therefore, it was possible to establish a dependence of biodegradability on silica particle size. The ZP of A200 particles was less negative than the ZP of A380 particles, revealing the greater stability of the smallest nanoparticles. On the other hand, the TEM image of A380 silica nanoparticles used in this study corroborated the aggregation phenomenon that has a direct effect on even minor biodegradation.

**Fig. 5:**
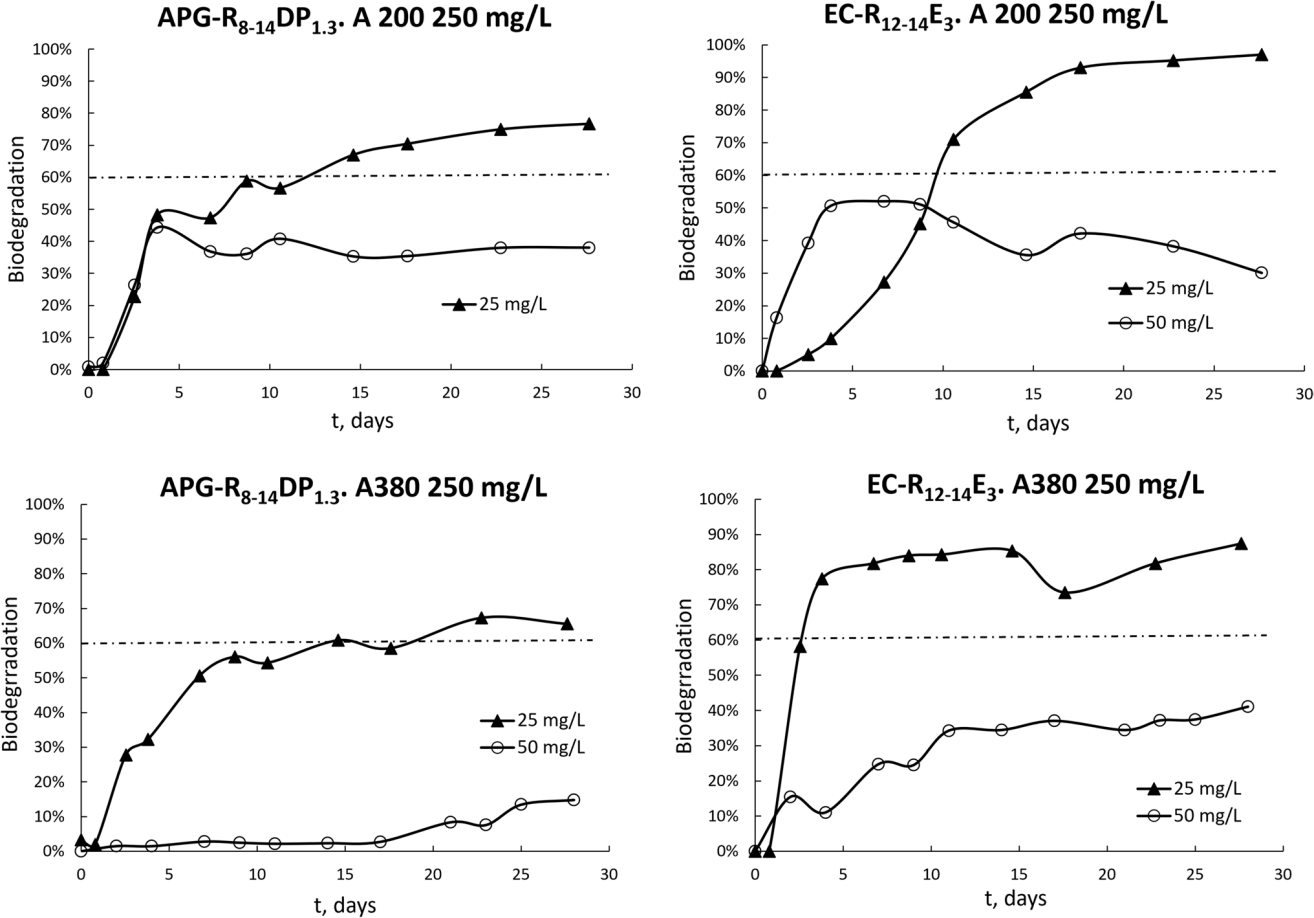
Influence of surfactant concentration on biodegradation profiles

The influence of initial surfactant concentration on biodegradability was demonstrated (Fig. 5). In general, surfactants biodegrade more easily at lower initial concentrations in the presence or absence of nanoparticles, which was the case for the two surfactants studied here. The minimum level of 60% ultimate biodegradation after 28 days was not reached for anionic and nonionic surfactants when the initial concentration of surfactant was 50 mg/L in the presence of nanoparticles, regardless of their size. This phenomenon is reflected in the characteristic parameters calculated for the biodegradation profiles, such as V_M_, t_L_, t_½_, and B. Therefore, the average velocity of biodegradation V_M_ and biodegradability B was greater for lower initial concentrations, and the latency time t_L_ and half-life time t_½_ were greater.

For nanofluids containing A200, the reduction in biodegradability, which was attributed to an increase in surfactant concentration, was more pronounced for the nonionic surfactant.

### Biodegradability of surfactant-nanoparticle mixtures

The biodegradabilities of anionic/nonionic surfactant mixtures were evaluated to understand the interactions and synergies among different kinds of surfactants. Surfactants are used as cosurfactants in many formulations, and therefore, the ecotoxicological and interfacial interactions in binary mixtures with a 1:1 weight ratio of ether carboxylic derivative surfactants and alkyl polyglucosides were investigated. Mixtures of surfactants in detergents, household care products and industrial formulations generally include nonionic/nonionic, cationic/cationic, anionic/anionic, and amphoteric/amphoteric surfactant pairs. However, it has been demonstrated that the synergistic effects between them increase with increasing charge difference (Werts and Grady 2011), meaning that synergisms between nonionic/nonionic or anionic/anionic pairs are less than those between nonionic/anionic surfactants (Kume et al. 2008).

**Fig. 6:**
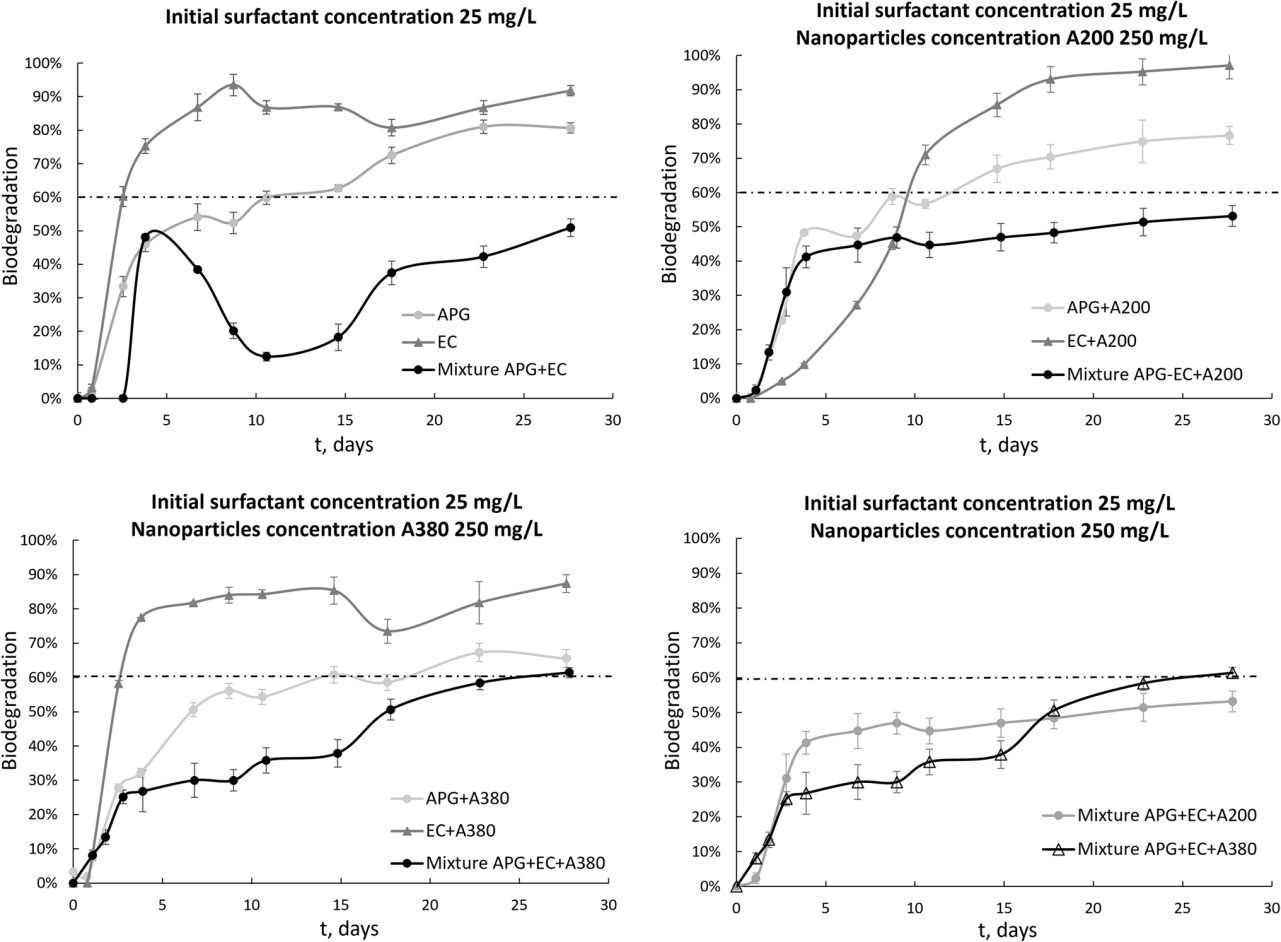
Biodegradation profiles for binary mixtures of surfactants and nanoparticles

The level of biodegradation for APG-EC binary mixtures is lower than those for solutions with single surfactants. This negative synergistic effect may be explained by reductions of the electrostatic repulsions between the head groups of anionic surfactants upon inclusion of nonionic head groups, which results in lower aggregate stability and thus an increase in CMC for binary mixtures of anionic-nonionic surfactants. This occurs both in the presence and absence of nanoparticles (Fig. 6).

Data for binary mixtures indicated that the lowest biodegradation level appeared when a mixture formed by the surfactant APG-EC and larger nanoparticles was tested. These results may suggest formulations of commercial surfactant mixtures with augmented biodegradability, especially if the surfactants EC and APG are incorporated.

## Conclusions

This work investigated whether silica nanoparticles enhance the biodegradability of surfactants and other surfactant properties, particularly interfacial and adsorption behaviours. Binary mixtures of nonionic and anionic surfactants were also investigated. The nonionic and anionic surfactants studied (APG and EC, respectively) decreased the surface tensions of air–water interfaces. The inclusion of negatively charged hydrophilic silica nanoparticles reduced the efficiency of the nonionic surfactant and considerably increased its CMC, but the effect was opposite for the case of the anionic surfactant. Increasing concentrations of surfactant and nanoparticles in the test medium resulted in decreases in the relative maximum mineralization for both surfactants. These results imply that surfactants assayed at low concentrations may be considered safe for the environment when formulated as nanofluids with or without nanoparticles and with an initial surfactant concentration of 25 mg/L. Measurements of binary mixtures indicated that the mixture with the lowest biodegradability was formed with the surfactant APG-EC and larger nanoparticles. Since biodegradation is the main mechanism for removing organic compounds, knowledge of the biodegradability of surfactants in combination with other additives is necessary for understanding the environmental behaviour of these mixtures before designing a detergent formula. These results can lead to a useful methodology for development of more biodegradable formulations.
